# The Impact of the Psychiatry Medical Student Scholars Program

**DOI:** 10.1007/s40596-024-02006-5

**Published:** 2024-07-10

**Authors:** Wan Y. Kwok, Niki Moftakhor, Peirce Johnston, Brian Evans, Melissa DelBello

**Affiliations:** 1 University of Illinois Chicago, Chicago, IL USA; 2 Ascension St. John Children’s Hospital, Detroit, MI USA; 3 University of Cincinnati College of Medicine, Cincinnati, OH USA

**Keywords:** Medical education, Research, Psychiatry education, Medical students

## Abstract

**Objective:**

Providing medical students with psychiatry research opportunities early in their careers may contribute to fostering career interests and increasing research literacy and interest. In this report, the authors describe the Psychiatry Medical Student Scholars Program (MSSP) at the University of Cincinnati College of Medicine and the results from a survey of its impact on student career outcomes over 9 years.

**Methods:**

All MSSP participants were invited to complete an online survey via SurveyMonkey to assess the impact of the program on their interest in psychiatry and research.

**Results:**

The MSSP began in 2012 with one student. There have been 47 MSSP participants from 2012 to 2021. Rapid growth of the MSSP was seen with class sizes ranging from 1 to 11. At the time of survey, nineteen MSSP alumni graduated medical school and 28 were still in medical school. Sixty-six percent of eligible participants responded, with a 53% response rate for medical school alumni and a 75% response rate for current medical students. Nine out of nineteen (47%) MSSP students who had graduated from medical school selected a career in psychiatry. Eighty-four percent of participants had presented or published their research. Sixty-eight percent of participants reported that the program has been valuable in deciding their future specialty.

**Conclusions:**

Participants tended to credit exposure to psychiatric research as medical students with fostering interest in the field and aiding in their career decisions. The components of the program described can be replicated at other institutions to increase exposure to psychiatric research.

The field of psychiatric research has greatly improved our understanding of mental illnesses. However, the number of psychiatrists pursuing a research career has been declining [1–3]. Many efforts have been made to increase research training of psychiatry residents [4, 5] and of attending physicians [6, 7]. However, early exposure to psychiatric research among medical students has received less attention.

Broadening beyond the need for psychiatrist-researchers, the need for psychiatrists continues to grow. One report found that 54% of counties in the United States had no psychiatrists [8]. Additionally, it is projected that in 2024 there will be a record shortfall of psychiatrists in the workforce due to population growth and retirement of over half of the workforce, with a low estimate shortage of 14,280 psychiatrists [9]. Fostering an interest in psychiatry for medical students, early in their career, is crucial to addressing the increasing need.

Programs that provide research opportunities, clinical exposure to medical specialties, and mentorship exist within many medical schools throughout the United States. However, few have been systematically evaluated for impact on outcomes. One review identified 20 reports of medical student research programs from 1950 to 2013, finding that overall research experiences were characterized as positive for both research skills and interpersonal skills [10]. Another survey of an NIH-sponsored research experience found that a majority of respondents felt that their mentor had positively influenced their choice of specialty, with participants more likely than their classmates to participate in research after medical school [11]. A review focused on medical student mentorship programs examined 14 papers from 2000 to 2008 and reported that mentorship programs were most effective in the context of research and career counseling [12]. However, there are few reports of medical student psychiatry research programs. A PubMed search identified only two articles on this topic. First, Masaki et al. [13], reviewed a virtual model summer psychiatry program for underrepresented medical students. Second, Stein et al. [14] reported the results of a survey of a multi-site program focused on Child and Adolescent Psychiatry that showed increased positive perception of mentorship towards career guidance and research after 1 year. With these considerations in mind, we examined the outcomes from the University of Cincinnati College of Medicine Psychiatry Medical Student Scholars Program (MSSP), a longitudinal program focused on incorporating a psychiatric research experience and mentorship. Additionally, we evaluated which components of the program were reported to be most helpful to the students in their careers.

## Methods

All MSSP participants were invited through email to participate in an anonymous survey via SurveyMonkey. Participants were sent two reminder emails 2 weeks apart and then a month following the initial email. Alumni who did not respond to emails or had non-working emails were contacted by telephone and/or new email addresses were identified through the internet. We were unable to obtain contact information for two alumni despite best efforts. Survey responses were collected for 4 weeks. All survey responses were anonymous. Eleven items were analyzed for both alumni and current students. The question formats consisted of multiple choice, choose all that apply, open response narrative, and Likert Scale.

The MSSP is a longitudinal program that spans 4 years of medical education, with an intensive research component taking place in the summer between the first and second year of medical school. The aim of this program is to help students obtain early exposure to the field of psychiatry, in general, and psychiatric research, specifically. Students apply to the Psychiatry and Behavioral Neuroscience MSSP after their first semester of medical school. There are several MSSP tracks throughout the University of Cincinnati College of Medicine including anesthesiology, cardiovascular medicine, child and adolescent health, emergency medicine, family medicine, geriatric health, integrative and lifestyle medicine, medical education, nephrology, neuroscience, pulmonary, and women’s health. Each track is independently run and varies greatly in activity requirements, availability of funding, and number of students accepted.

The Psychiatry MSSP provides a stipend for the summer period of research. Funds for the stipend are provided through philanthropy. Uniquely among the MSSPs available at the University of Cincinnati College of Medicine, there is no current limit on the number of students accepted. To be considered, students are required to be in good academic standing and to apply by submitting their resume and a personal statement about their research interests. Additionally, students must be available to complete the program in-person at the University of Cincinnati or Cincinnati Children’s Hospital. Every student who fulfills these requirements is accepted. Accepted students are then matched with mentors based on their indicated research interests. Research projects span a wide variety of topics across psychiatry, including mood, psychosis, addiction, suicidality, eating disorders, translational science, and pharmacology. Since the program’s beginning, thirty faculty have participated as mentors. Students complete a 5-week, full-time research experience. After the summer research experience, students are encouraged to present their research at the College of Medicine’s Annual Research Symposium as well as at local, state, and national conferences. Mentors also incorporate clinical experiences and career mentoring for their mentees. Depending on the project, students may continue to work on their research after the summer.

In addition to the summer research experience, there are other longitudinal components. Students meet with the director of medical education in psychiatry throughout their 4 years. Informal events with fellow MSSP participants were also arranged but were canceled or virtual during the 2020 and 2021 summers due to the COVID-19 pandemic. Students are required to attend 15 h of didactics, take an online Research 101 course, perform 10 h of service and leadership, and obtain 10 h of clinical shadowing. Didactics are fulfilled through grand rounds, journal clubs, and psychiatry interest meetings through the Department of Psychiatry and Behavioral Neuroscience. Students who successfully complete all requirements for the program are designated as Medical Student Scholars on their transcript and have this distinction announced during graduation.

## Results

There had been 47 Psychiatry MSSP participants from the classes of 2016 to 2024. The size of the program expanded, from one student in the original class of 2016 to 12 in the class of 2023. The average class size ranges from 160 to 175 MD students, with MSSP participants in the class of 2023 representing 7% of the class (12/171). From program census data, there were 19 MSSP participants who graduated from medical school and 28 participants in medical school at the time of the survey. The overall response rate to the survey was 66% (31/47), with a 53% (10/19) response rate from medical school alumni and a 75% (21/28) response rate from current students. At the time of the survey, nine out of nineteen (47%) MSSP alumni who graduated from medical school matched into a psychiatry residency program. Of the alumni participants who responded to the survey, seven of the ten (70%) matched psychiatry.

Of the respondents, 48% (15/31) identified as women, 45% (14/31) identified as men, and 6% (2/31) identified as gender non-binary. Of current students, 57% (12/21) identified as women, whereas of alumni 30% (3/10) identified as women. Of current student respondents, 29% (6/21) were in their second year of medical school, 33% in their third year (7/21), and 38% (8/21) in their fourth year. On average, alumni respondents graduated 2.8 years ago.

Current students were asked which aspects of the MSSP program motivated them to participate. One hundred percent were motivated due to interest in psychiatry as a career, 81% (17/21) were interested in research, 67% (14/21) stated they were motivated to be paid over the summer, and 62% (13/21) were motivated by the designation of medical student scholar.

A majority of participants, 68% (21/31), continued their research with their mentor past the dedicated summer research experience. Additionally, 84% (26/31) of participants presented their research through poster presentations, book chapters, and/or publications. For current students, 52% (11/21) had presented a poster, 33% (7/21) had submitted or published a paper, 14% (3/21) published a book chapter, and 48% (10/21) still had research in progress (Table 1). For alumni, 70% (7/10) presented a poster, 30% (3/10) published a paper, and 10% (1/10) published a book chapter. Forty percent (4/10) of alumni are engaged in research in their current position.

**Table 1:** Outcomes of the Psychiatry Medical Student Scholars Program (MSSP) at the University of Cincinnati College of Medicine

Variable	MSSP current medical students	MSSP alumni respondents
	*N* = 21	*N* = 10
Outcome of research experience		
Poster presentation	52%	70%
Paper submission/publication	33%	30%
Book chapter	14%	10%
None of the above	0%	10%
Research still in progress	48%	0%
How many research experiences do you currently have (poster presentations, journal articles, oral presentations)?		
None	10%	0%
One	14%	10%
Two to three	33%	30%
Four to six	23%	30%
Seven to nine	10%	10%
Ten or more	10%	30%
Did you continue research after the summer period?		
Yes	71%	60%
No	29%	40%

**Fig. 1:**
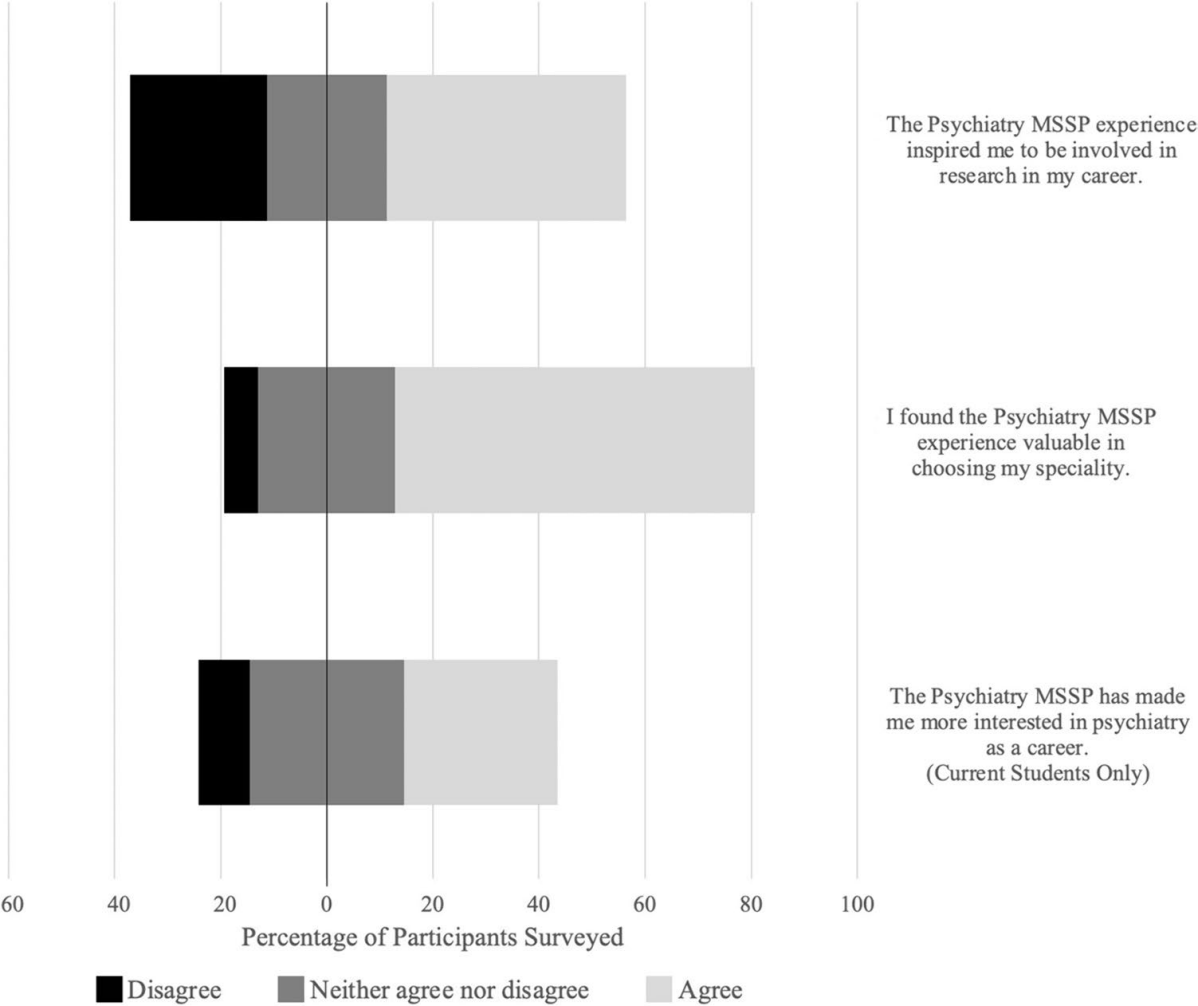
Distribution of Likert Scale responses from the Psychiatry Medical Student Scholars Program (MSSP) participants

All medical school graduates agreed that the MSSP experience was valuable in choosing their specialty. However, current students were less likely to agree, with 52% (11/21) selecting agree, and 10% (2/21) selecting disagree (Fig. 1). In narrative responses, participants were asked to describe the positive aspects of their MSSP experience. The most common theme was exposure to research with 65% (20/31) of respondents addressing this aspect, including identifying a project with built-in funding, exposure to psychiatric research, and learning to analyze data. The second most common theme was mentorship, with 61% (19/31) of respondents addressing their positive relationship with their mentor and other faculty.

Participants were surveyed for areas for improvement for the MSSP. Free response answers commonly commented on increased clinical psychiatry exposure, opportunities for networking, and mentors setting expectations. Current students were more likely to identify areas for improvement than alumni, specifically requesting increased student interaction and socialization. Forty percent of alumni stated there were no areas of improvement, in comparison to none of the current students.

## Discussion

A majority of the survey participants, 68% (21/31), were current medical students. This reflects the rapid growth of the Psychiatry and Behavioral Neuroscience MSSP over 9 years and the growing interest in psychiatry as a career. In summary, the Psychiatry and Behavioral Neuroscience MSSP provides early exposure to research in psychiatry resulting in posters and publications for the majority of participants. While all participants were interested in psychiatry at the start of the program, not all participants ended up pursing psychiatry as a specialty. This likely reflects the decision-making process of students as they proceed through clinical years of medical school. Additionally, most participants found it helpful in choosing their specialty, with increased agreement it was helpful in respondents who had completed the program. The components of the program described can be replicated at other institutions to increase exposure to psychiatric research.

One unanticipated result of this survey was the notable difference in responses between current students and alumni. In general, alumni may better be able to reflect on the entire course of their specialty decision in comparison to current medical students. Additionally, most of the fourth-year medical students were positive about their MSSP experience. The largest MSSP class was medical students who were in their third year at the time of the survey. For this class, the 2020 summer research experience was significantly altered due to the COVID-19 pandemic. Medical students were not permitted to be onsite, which meant fewer clinical experiences, and decreased onsite interactions with faculty and other MSSP participants. This unprecedented change in the switch to remote activities and increase in outside stressors impacted medical students and faculty alike.

This study is limited by the nature of it being a retrospective survey of participants from one program. While it may have been interesting to compare the psychiatry MSSP to other similar programs, each program is independently run and operates differently. The process by which students are accepted to MSSP, funding availability, required activities, and structure may all impact outcomes. Additionally, no comparison group was available. Possible comparison groups for future evaluation of similar programs might include students initially interested in psychiatry who did not participate, or students who applied to psychiatry residency without participating in the MSSP. This survey is also subject to response bias, i.e., those who responded were more likely to have pursued a career in psychiatry and have had a positive experience. Alumni who responded to the survey were more likely to have gone into psychiatry than the average alumni participant. Participants in the University of Cincinnati College of Medicine Psychiatry MSSP tended to credit exposure to psychiatric research as medical students with fostering interest in the field and aiding in their career decisions.

## Data Availability

Data is not available in order to protect privacy of participants in the study.
